# Simultaneous photoadhesion and photopatterning technique for passivation of flexible neural electrodes based on fluoropolymers

**DOI:** 10.1038/s41598-020-78494-w

**Published:** 2020-12-07

**Authors:** Yong Hee Kim, Sang-Don Jung

**Affiliations:** ICT Creative Research Division, Electronics and Telecommunications Research Institute, 218 Gajeong-ro, Yuseong-gu, Daejeon, 34129 Republic of Korea

**Keywords:** Biomedical engineering, Polymers

## Abstract

Herein, we introduce a method to simultaneously photoadhere a photocrosslinkable polymer to a plasma-treated fluoropolymer while photopatterning the photocrosslinkable polymer via a single-photo-exposure as a new electrode passivation technique. Photoadhesion was determined to result from plasma-generated radicals of the plasma-treated fluoropolymer. Crystallinity of the fluoropolymer was analysed to determine the photoadhesion strength through its effects on both the formation of radicals and the etching of fluoropolymers. Passivation feasibility of simultaneous photoadhesion and photopatterning (P&P) technique were demonstrated by fabricating an Au electrocorticography electrode array and modifying the electrode with electro-deposited metallic nanoparticles. Adhesion of sputter-deposited Au to the fluoropolymer was dependent on mechanical interlocking, indicated by the formation of Au clusters which are typically influenced by the surface temperature during the sputter-deposition and the glass transition temperature of the fluoropolymer. The adhesion of Au to the fluoropolymer without an additional adhesion promotor and the proposed P&P passivation technique would help prevent detachment of the electrode and the delamination of the passivation layer in fluoropolymer-based neural electrode.

## Introduction

Flexible neural electrodes based on polymeric substrates exhibit several advantages over those based on inorganic substrates, including that they do not break easily under mechanical stress and that they can accommodate natural organ curvatures. These polymer-based electrodes offer reduced mechanical mismatch against neural tissues due to the relatively low Young’s modulus of the polymeric substrate when compared to that of conventional Si. This low Young’s modulus alleviates foreign-body responses classified as biotic mode failures^1–4^. Although many flexible neural electrodes have been developed as in vivo interfaces with neural tissues, they still suffer from abiotic modes of failure (e.g*.* electrode corrosion or delamination of a passivation layer^5,6^). Due to the inherently weak interactions between typical electrode metal (Au or Pt) and polymer layers, a chromium or titanium layer is routinely introduced to promote adhesion between these layers. However, these adhesion-promoting layers may be dissolved by abundant alkaline metal ions and oxygen radicals that exist within neural tissues and cerebrospinal fluid and are released by immune cells, respectively; exposure to these endogenous chemical species ultimately delaminates the layered electrode. Water molecules that are absorbed or diffused within the substrate or passivation layer may also contribute to delamination by both facilitating the diffusion of alkaline metal ions and altering structural and physical properties^7^.

**Figure 1:**
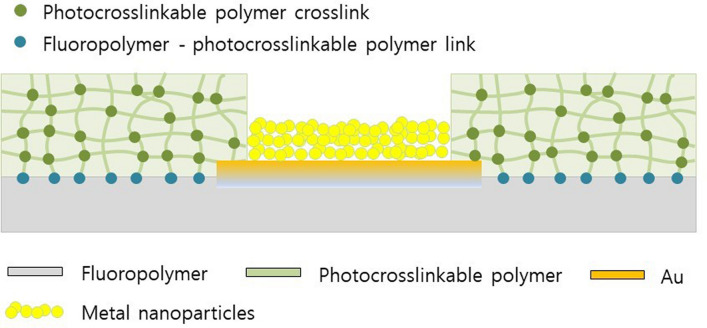
Schematic representation of the P&P electrode passivation process based on a photocrosslinkable polymer and plasma-treated fluoropolymer composite system.

To achieve a flexible neural electrode free of abiotic mode failures, we recently fabricated a fluoropolymer-based flexible neural electrode, where fluoropolymers were used both as the substrate and passivation layer^8^. The fabricated fluoropolymer-based neural electrode consisted of a chemically stable and biocompatible fluoropolymer and an Au electrode; this composite does not require an adhesion-promoting metal layer and is chemically stable. During fabrication, this fluoropolymer-based electrode was passivated by thermally pressing plasma-treated fluoropolymer films followed by plasma etching of the fluoropolymer passivation layer to expose an active electrode area. Covalent bonding of plasma-generated radicals was proposed as a likely mechanism accounting for the co-adhesion of fluoropolymer films; plasma-generated radicals have been utilised as an underlying source of grafting copolymerisation-based surface modifications onto fluoropolymers. Herein, we attempted to apply photocrosslinkable polymers as a passivation layer, replacing plasma-treated fluoropolymer passivation films; this was done to extend the modification capabilities of grafting copolymerisation-based surfaces to electrode passivation films. The photocrosslinkable polymers were anticipated to be photo-chemically bound to a fluoropolymer surface via the formation of covalent bonds between photo-generated radicals on photocrosslinkable polymers and plasma-generated radicals on fluoropolymers. Photocrosslinkable polymers can act as negative-tone photoresists, facilitating both covalent photoadhesion and generating the exposure of active electrode areas during a single photo-exposure process, as depicted in Fig. 1; these capabilities would allow the circumvention of both the thermal pressing and the plasma etching steps involved in previous fluoropolymer-based neural electrode fabrication methods.

Herein, we demonstrate a novel photoadhesion and photopatterning (P&P) technique by adopting SU-8 as the photocrosslinkable polymer and various fluoropolymer substrates, including fluorinated ethylene propylene (FEP), perfluoroalkoxy alkane (PFA), and polytetrafluoroethylene (PTFE). SU-8 is a commonly used biocompatible epoxy-based negative photoresist during the fabrication of microelectromechanical system-based neural electrode arrays^9–14^ and during electrocorticography (ECoG) interfaces^15–18^. We also investigate the photoadhesion processes in terms of adhesion strength (AS), radical density, and surface morphologies. Additionally, we examine both the adhesion mechanism and adhesion enhancement present in the Au and fluoropolymer composite system by investigating surface roughness effects in terms of both the AS and interfacial distribution of Au clusters. When combined with the Au-fluoropolymer adhesion enhancement, the passivation ability of our new simultaneous P&P technique was demonstrated via the fabrication of an ECoG Au electrode array. Additionally, the passivation performance of the fabricated electrodes was evaluated by modifying the Au electrode with electro-deposited metallic nanoparticles (NPs).

## Results

### Simultaneous photoadhesion and photopatterning

After the photopatterning of SU-8 layers which have undergone spin-coating on both the plasma-treated and non-treated FEP film surfaces, the AS of SU-8 test patterns to FEP was checked with Scotch^®^ tape (810D, 3 M). As expected, the SU-8 test patterns adhered to plasma-treated FEP were not peeled off (Supplementary Fig. 1a); however, the SU-8 test patterns on non-treated FEP were entirely peeled off (Supplementary Fig. 1b). This quick test qualitatively demonstrated the utility of the proposed P&P technique based on a composite system comprised of SU-8 and FEP. To quantitatively investigate the P&P technique, a metal stud was adhered to the SU-8 test pattern; the AS between this stud and the SU-8 was then measured by a tensile pull test. A typical load-strain curve is provided as Supplementary Fig. 2. The AS of the SU-8 test pattern with non-treated fluoropolymers was approximately zero, neglecting the effects of the over-pasted epoxy binding the metal stud and SU-8 test pattern.

**Figure 2:**
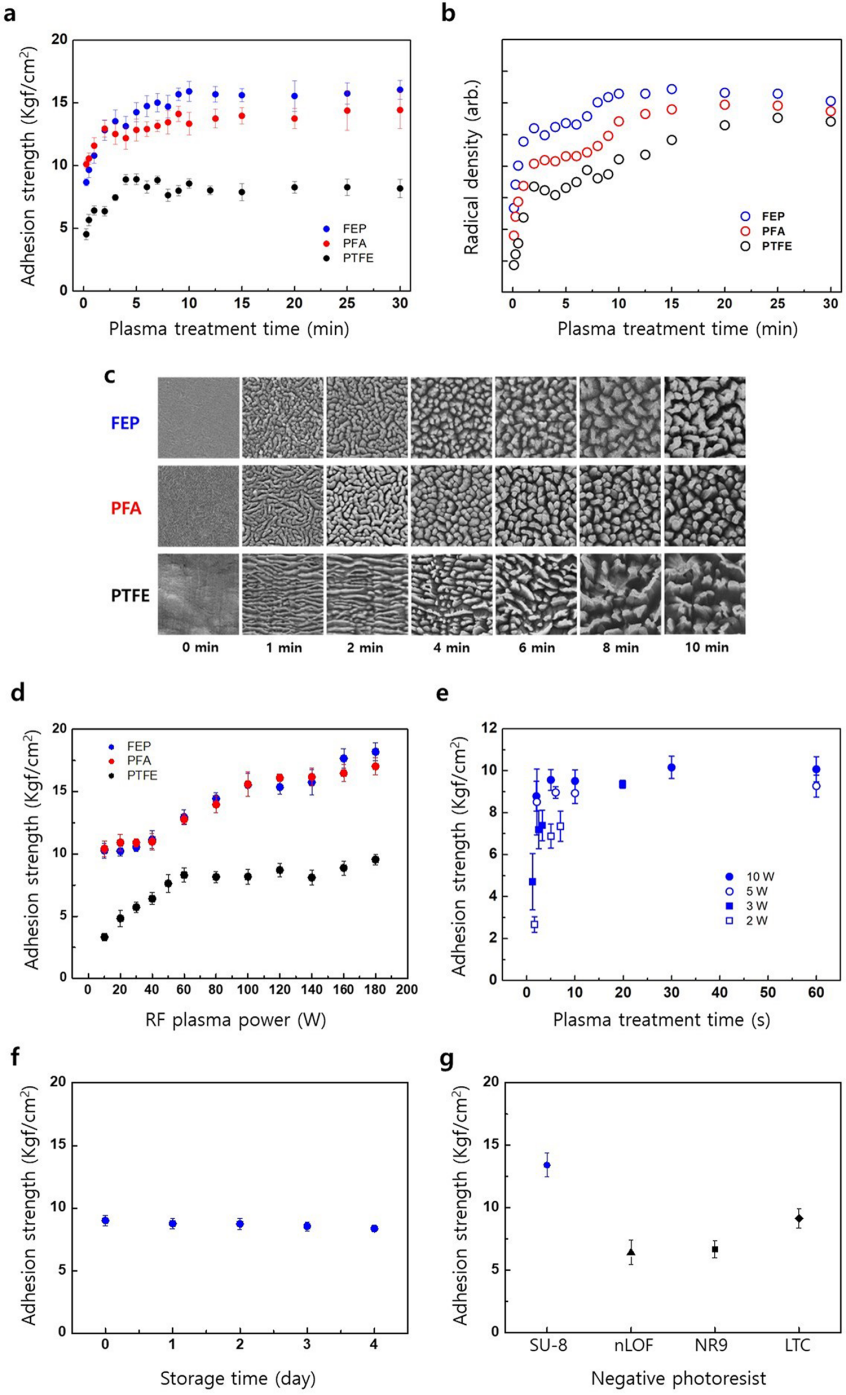
Effects of Ar RF plasma treatment (RF power: 40 W, working pressure: 15 mTorr) time (**a**) on the AS between SU-8 and fluoropolymers and (**b**) on the radical density of fluoropolymers. (**c**) FESEM surface images of FEP (top), PFA (middle), and PTFE (bottom) after various plasma treatment times. The dimension of each image is 2 μm × 2 μm. (**d**) Effect of Ar RF plasma power on the AS between SU-8 and fluoropolymers (plasma treatment time: 1 min). (**e**) The AS between SU-8 and plasma-treated FEP under milder conditions and plasma treatment times. Plasma treatment conditions are listed in the legend. (**f)** The AS between SU-8 and plasma-treated FEP (RF power: 10 W, plasma treatment time: 1 min) with respect to the storage time in units of days. (**g)** The AS of commercial negative photoresists with plasma-treated FEP (RF power: 40 W, RF plasma treatment time: 4 min). nLOF, NR9, and LTC denotes AZ nLOF 2070 (AZ), and NR9-3000PY (Futurrex), and LTC 9520 (FujiFilm), respectively.

Generally, the electrons and free radicals created within the plasma collide with the polymer surface and cleave covalent bonds, resulting both in radical generation and chemical etching. Therefore, the photoadhesion of SU-8 to fluoropolymers can be mechanistically accounted for by both covalent bonding between photo-generated radicals on SU-8 and those of the plasma-treated fluoropolymers and mechanical anchoring due to the etched surfaces. To clarify the effects of surface morphology on the photoadhesion between SU-8 and fluoropolymers in terms of AS, we observed the results of plasma treatment conditions, such as RF power and plasma treatment time, on the adhesion between SU-8 and fluoropolymers (Fig. 2).

Figure 2a and b show the effect on the adhesion of SU-8 to fluoropolymers of the plasma treatment time and the radical density of fluoropolymers, respectively. Overall, the AS and the radical density exhibit similar behaviours to the plasma treatment time. Both properties increased rapidly up to 2 min before increasing slowly after the 2 min mark. The full field emission scanning electron microscopy (FESEM) images of the FEP film within 1 min of plasma-treatment are shown in Supplementary Fig. 3, representing a clear lamella structure even at 15 s. The AS between the SU-8 and FEP surfaces is slightly higher than that between SU-8 and PFA surfaces and approximately two times higher than that between SU-8 and PTFE surfaces. The AS between SU-8 and FEP, PFA, and PTFE exhibited saturation behavior at longer plasma treatment times. The order of magnitude of the radical density displays similar reductions as the AS; although the radical density of PFA is incomparable to that of FEP, it is positioned between FEP and PTFE. The radical density of fluoropolymers also exhibited saturation behavior at longer plasma treatment time. As shown in Supplementary Fig. 4, the electron spin resonance (ESR) spectrum of FEP, PFA, and PTFE all exhibit a slight asymmetric singlet line with an average *g* value of 2.015, 2.016, and 2.017, respectively; these values are similar to the published values of 2.016^19^, 2.016^20^, and 2.017^20^ for the air-exposed radicals of FEP, PFA, and PTFE, respectively. The published values and observed singlet spectral features were attributed to the formation of peroxy radicals. As shown in Fig. 2c, FESEM surface images of all fluoropolymers exhibit transition from a lamella to a protrusion structure with an increasing plasma treatment time. The faint lamella morphology of untreated FEP and PFA and the lamella-free morphology of untreated PTFE indicate that the outermost surface of fluoropolymers is dominantly covered with amorphous phase. Notably, the actual radical density for longer treatment times would be much smaller than either the apparent radical density or the geometrical radical density due to the roughened surface. To confirm the chemical structure effects on the adhesion between SU-8 and fluoropolymer, the ASs were measured between SU-8 and FEP, PFA, and PTFE under a wide range of RF powers. As shown in Fig. 2d, the ASs of SU-8 to both FEP and PFA are again comparable and found to be two-fold higher than that of SU-8 to PTFE.

**Figure 3:**
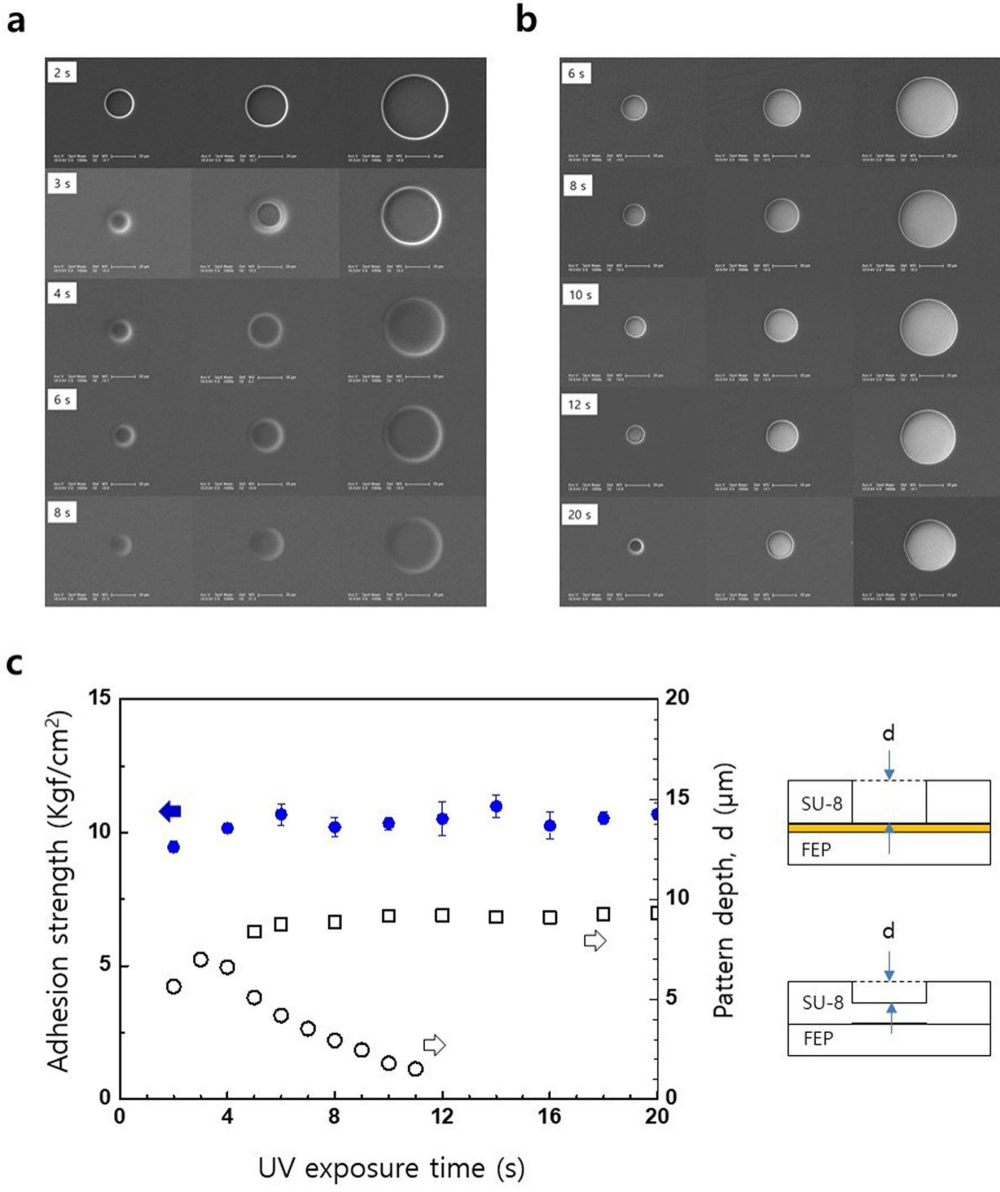
FESEM images of 20-μm (left), 30-μm (centre), and 50-μm (right) SU-8 patterns photopatterned on plasma-treated FEP (@ 20 W, 1 min) (**a**) and on an Au layer (**b**) with respect to UV exposure time. (**c**) The AS between SU-8 and FEP (closed circle in blue), the pattern depth of SU-8 on Au layer (open rectangle), and that of SU-8 on FEP (open circle) with respect to UV exposure time. Pattern depth data were obtained from 50-μm line patterns via step profilometer (Alpha-step 500, KLA-Tencor). Multiplying the UV exposure time by the power density (22.7 mW/cm^2^ @ 365 nm) yields the exposure dose in units of mJ/cm^2^.

**Figure 4:**
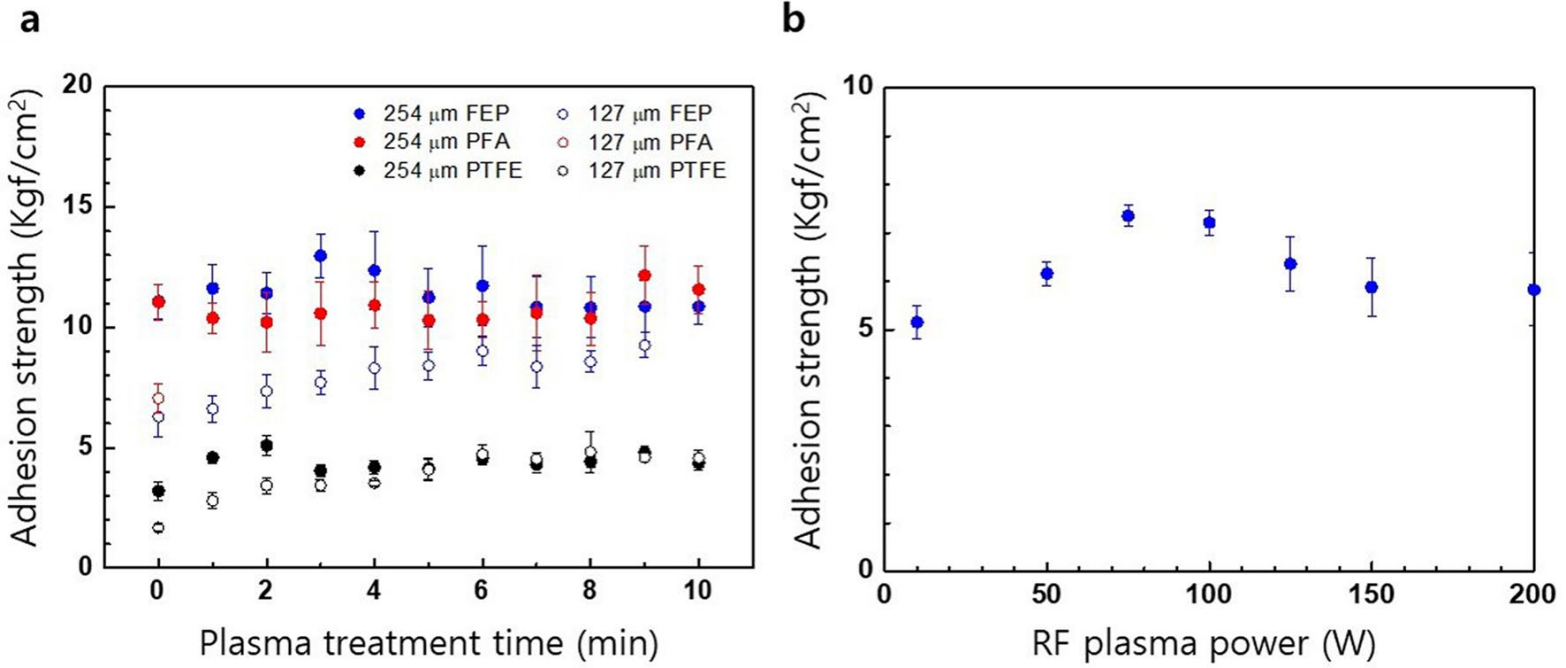
(**a**) The AS between Au and fluoropolymers relative to both the plasma treatment time and the fluoropolymer film thickness. Fluoropolymers were treated at an RF power of 40 W under working pressure of 15 mTorr. (**b**) Effect of RF sputtering power on the AS between Au and a non-treated 127-μm thick FEP film. RF sputtering power was applied in a two-step mode. Up to a nominal thickness of 20 nm, RF power in the range of 20–200 W was applied; afterward the RF power was switched to 50 W and maintained up to a nominal thickness of 200 nm. The RF sputtering power denotes that applied during the first step.

The exposure of an electrode to plasma during our P&P passivation process is unavoidable. Therefore, milder plasma treatment conditions are preferred to reduce any plasma-induced damage to the electrode. The effect of milder plasma treatment conditions –such as a lower RF power or a shorter treatment time – on the adhesion properties was investigated and the results are provided as Fig. 2e. No significant changes to the AS were observed with respect to the plasma treatment time when the RF power is greater than 5 W. However, AS values below 5 Kgf/cm^2^ were obtained both at lower RF powers (2 W and 3 W) and at very short treatment times (1–2 s). These results indicate that exposure to an RF power of 5 W for only a few seconds is sufficient to generate radicals along the sample surface. As shown in Supplementary Fig. 5, an FESEM image of a FEP surface after plasma treatment at 2 W for 2 s does not show appreciable changes when compared to an untreated surface.

**Figure 5:**
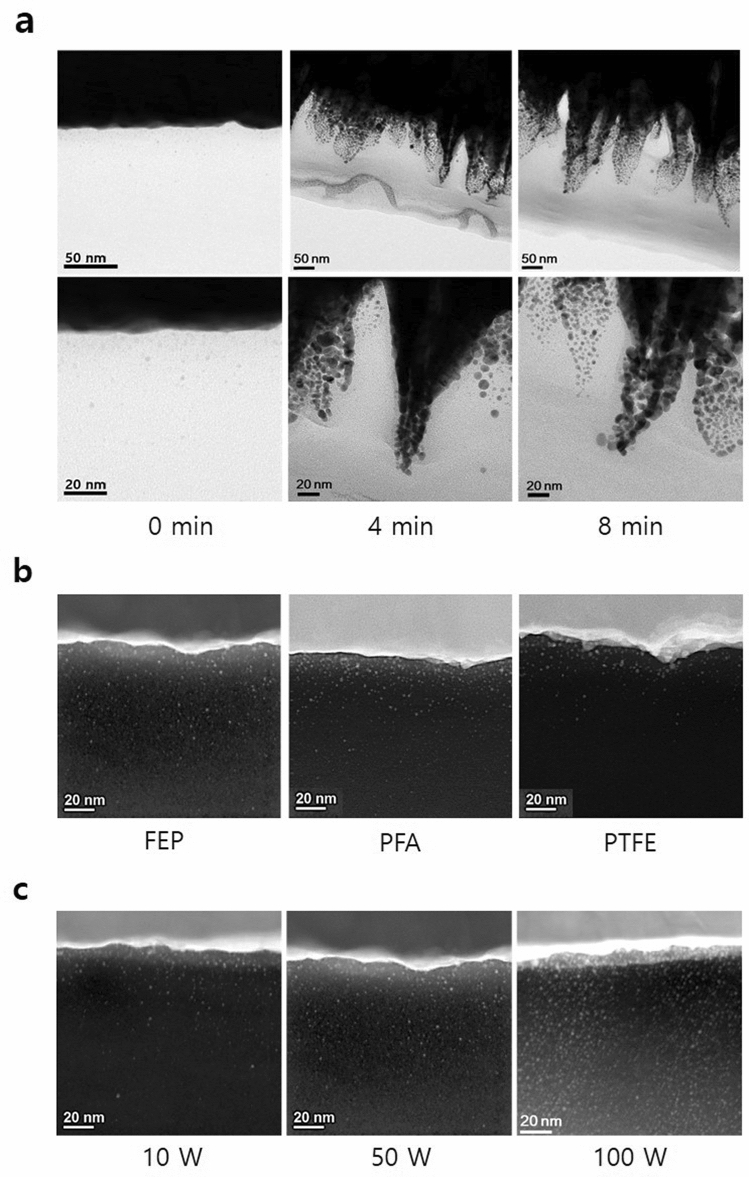
(**a**) Cross-sectional transmission electron microscopy (TEM) images of the Au-FEP interface with respect to plasma treatment time. The FEP was plasma treated at an RF power of 40 W, and Au was deposited at an RF sputtering power of 50 W. (**b**) Cross-sectional STEM images of the Au-FEP, Au-PFA, and Au-PTFE interfaces. Fluoropolymers did not undergo plasma treatment, and Au was deposited at an RF sputtering power of 50 W. (**c**) Cross-sectional STEM images of an Au-FEP interface with respect to the RF sputtering power. All images are of a 127-μm thick FEP film.

Information on either the lifetime or decay characteristics of plasma-generated radicals is highly useful for generation of a fabrication schedule. To determine the lifetime of plasma-generated radicals, we monitored the AS between SU-8 and FEP after plasma treatment, instead of by ESR measurements^21,22^. To conduct this measurement, plasma-treated FEP samples were stored in a fluoroware wafer carrier and then placed on a clean bench under a maintained humidity and temperature < 35% and 24 ± 1 °C, respectively. Ambient light was blocked to eliminate any effects caused by environmental photons. As shown in Fig. 2f., the monitored AS decreased by 5% after four days of storage.

To widen the range of possible photocrosslinking polymer choices, we employed several commercially available negative photoresists, including AZ nLOF 2070 (AZ), NR9-3000PY (Futurrex), and LTC 9520 (Fujifilm). We checked both the photopatterns and photoadhesion in terms of the measured AS. As shown in Fig. 2 g, all negative photoresists formed test patterns and exhibited photoadhesive abilities, even though the measured AS varied for the different negative photoresists employed. This result widens the range of applicable photocrosslinking polymer choices for use within our procedure, where each polymer provides unique physicochemical and photochemical properties.

### Photopatterning fidelity

To check the photopatterning fidelity of a SU-8 and FEP pair, SU-8 was photopatterned on both the plasma-treated FEP surface and the 200 nm-thick sputter-deposited Au surface. Round photomask patterns with diameters as small as 20 μm were applied to reflect the real passivation characteristics of a neural electrode. The AS was also measured with respect to UV exposure time, measuring effects of UV exposure dosage. Figures 3a and 3b show the opened SU-8 patterns photopatterned on both the FEP and Au surfaces, respectively. As shown by the top FESEM image of Fig. 3a, the SU-8 photopatterns on FEP exhibit clear circular patterns with excellent fidelity after a 2 s exposure time, while maintaining robust adhesion between SU-8 and FEP surfaces. At an exposure time of 3 s, smooth edges form due to UV reflection from the FEP surface; these edges partially cover the bottom. As the exposure time is increased beyond 3 s, the smooth edges widen and become the dominant feature covering the bottom; at longer exposure times, the patterns become fuzzy and obscure. As shown in Fig. 3c, the pattern depth of a SU-8 pattern on FEP (open circle) decreased with an increasing exposure time. This decrease would be resulted from the reflection of incident UV followed by wave-guiding through SU-8 layer due to the higher refractive index of SU-8 (1.668 at 365 nm^23^) than that of FEP (1.412 at 355 nm^24^). However, as shown in Fig. 3c (closed circle in blue), the AS between SU-8 and FEP is insensitive to UV exposure time, even for short time exposures of 2 s.

The FESEM images (Fig. 3b) and depth data (open rectangle, Fig. 3c) for SU-8 patterns on the Au layer exhibit excellent pattern fidelity over a wide range of exposure times. However, the diameter of the patterns decreased as the exposure time was increased; the circular patterns became distorted at higher expose times. As the AS is insensitive to exposure time, a shorter exposure time is advantageous for obtaining higher fidelity SU-8 patterns on either the FEP or an Au layer; yet, shorter exposure times inevitably result in diminished crosslinking, leading to a diminished pattern depth or film thickness, as shown in Fig. 3c.

### Au-fluoropolymer adhesion enhancement

The robust adhesion of a noble metal to a fluoropolymer without the use of an adhesive metal (*e.g.* Ti or Cr) is very critical to achieving abiotic issue-free neural electrodes. The interaction between noble metals and polymers is generally very weak due to higher cohesive energies of the metal compared to that of polymers. Therefore, we expect very poor adhesion at the Au and fluoropolymer interface due to their chemical inertness. However, there have been reports of successful Au deposition on fluoropolymers. Kim et al.^25^ reported a higher peel strength of *e*-beam evaporated Au on FEP compared to that of Au on PTFE; this finding was attributed to the high concentration of carbon sites at FEP with three fluorine neighbours, namely Au-carbon interactions. Successful adhesion of evaporated Au onto plasma-treated PTFE has also been reported and applied during the fabrication of neural electrodes^26,27^. Mechanical interlocking or anchoring due to plasma-induced surface roughening has been claimed to be responsible for the adhesion enhancement^28,29^. However, the role of plasma treatment is still unclear as it is not effective for all polymers^30,31^. To clarify the role of plasma treatment and reveal the adhesion mechanism of Au on fluoropolymers, we investigated the effect of plasma-treatment and Au sputtering conditions in terms of the AS, the interfacial morphology, and the cross-sectional distribution of Au clusters.

Figure 4a shows the AS of Au with fluoropolymers respective to the Ar plasma treatment time and the film thickness. For 254-μm thick films, the ASs coupling Au to both FEP and PFA are comparable and approximately double that between Au and PTFE. The ASs between Au and all fluoropolymers were not significantly influenced by the treatment time. Finally, for 127-μm thick films, the AS between Au and FEP is also higher than that between Au and PTFE. Initially, the AS of 127-μm thick FEP and PTFE films were lower than that of 254-μm thick films, approaching that of thick films after having undergone longer treatment time.

Contrary to the SU-8 and fluoropolymer composite, the AS between Au and non-treated fluoropolymers exhibited reasonable values, implying that the adhesion of sputter-deposited Au onto fluoropolymers is not caused by plasma-generated radicals. Plasma-treated 127-μm thick PFA films were torn off during the measurement of the AS, except for the non-treated case; plasma-treated FEP and PFA films thinner than 127-μm were torn off. As shown in Fig. 2c, Ar RF plasma treatment is accompanied by the etching of fluoropolymers. The typical etched trench profile of FEP, PFA, and PTFE is represented in Supplementary Fig. 6. Considering the almost identical etch depth for both FEP and PFA samples and the same order of magnitude for the tensile modulus (PFA < FEP < PTFE, Supplementary Table 1) the tearing of plasma-treated 127-mm thick PFA samples is contributed to by the synergetic effects of plasma-induced etching and relatively low tensile modulus of PFA.

**Figure 6:**
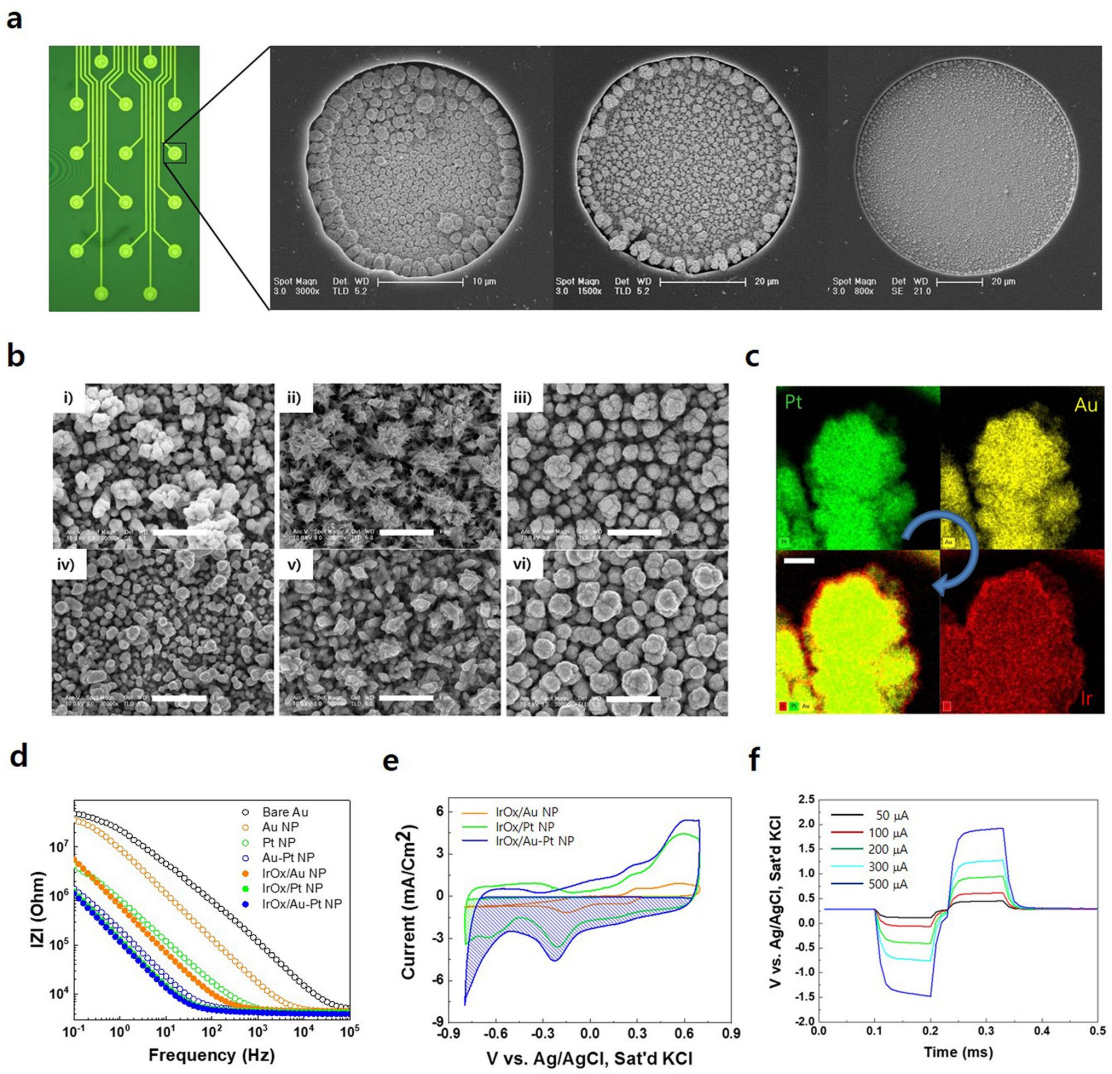
(**a**) Optical microscopy image of the SU-8 passivated ECoG electrode array and FESEM images of 25 μm, 50 μm and 100 μm electrodes modified with metallic NPs. (**b**) FESEM image of (i) Au NPs, (ii) Pt NPs, (iii) Au-Pt NPs, iv) IrOx/Au NPs, v) IrOx/Pt NPs, and vi) IrOx/Au-Pt NP-modified electrode. Scale bars denote 1 μm. (**c**) Cross-sectional TEM images of the Au-FEP interface with respect to plasma treatment time. Scale bar denotes 100 nm. (**d**) Electrochemical impedance spectroscopic results for the metallic NP- and IrOx/metallic NP-modified electrodes. (**e**) Cyclic voltammetry (CV) results for IrOx/metallic NP-modified electrodes. (**f**) Typical voltage transient of the IrOx/Au-Pt NP-modified electrode with an increasing biphasic current pulse amplitude (100 μs duration and 30 μs interpulse delay).

As shown in Fig. 4b, the AS between Au and the 127-μm thick FEP displayed a parabolic maximum between RF sputtering power of 75–100 W, decreasing at powers both below 75 W and above 100 W. When the RF sputtering power is low, the increase in substrate surface temperature is small, limiting diffusion into the bulk phase, while the probability of Au atoms penetrating the polymer bulk increases due to delayed full coverage of Au layer. Conversely, when the RF sputtering power is too high, the rapid coverage of the Au layer limits penetration of Au atoms into the bulk polymer, while the diffusion of penetrated Au atoms is facilitated. These limitations contribute to a decrease in the AS for RF sputtering powers both below 75 W and above 100 W.

As seen in Fig. 5a, TEM images of the non-treated cross-section exhibit a flat interface featuring clusters distributed along the flat interface. The initially flat cross-sectional interface transformed to a conical nanoprotrusion structure after extended treatment times; this transition was accompanied by an increase in the peak-to-valley height and an accumulation of enlarged Au clusters present in the valleys. It can be seen from the cross-section STEM images of non-treated FEP, PFA, and PTFE (Fig. 5b) that additional Au clusters are formed in both FEP and PFA bulk compared to within the PTFE bulk; furthermore Au diffused deeper into the FEP and PFA bulks than into the PTFE bulk. As evident in Fig. 5c, the amount of Au clusters formed in the non-treated FEP bulk increased with an increase in the RF sputtering power.

### Fabrication of ECoG electrode array and surface modification

To demonstrate the passivation potential of our new simultaneous P&P technique during the fabrication of a neural electrode, we designed and fabricated an in vivo 16-channel epidural ECoG electrode array by using a SU-8 and a 127-μm thick FEP film as the passivation layer and substrate, respectively. Because the surface roughness does not affect the adhesion of Au to fluoropolymers, and long-term Ar RF plasma treatment damage the Au electrode, we performed Ar plasma treatment at an RF power of 20 W for 1 min prior to both the sputter-deposition of Au and the passivation process using SU-8 3010. The unit fabrication processes are schematically represented in detail in Supplementary Fig. 7. Figure 6a conveys the optical microscopy and FESEM images of the fabricated ECoG electrode array. To verify the passivation performance of the photoadhered SU-8–FEP composite, we modified the exposed Au electrode surface with electrodeposited metallic NPs, including Au NPs, Pt NPs, and Au-Pt (1:4) NPs. As shown in Fig. 6a, the electro-deposited NPs were well-confined within the photodefined openings with 25, 50, and 100 μm diameters. These results demonstrate that our newly developed P&P technology possesses a sufficient passivation capability during the fabrication of neural electrodes. The passivation and electrodeposition of an electrode with an opening diameter of 25 μm implies that the dual-function photoadhesion and photopattering technique on the SU-8 and plasma-treated FEP composite can be utilized to fabricate intracortical multi-electrode arrays (MEAs).

As shown in the magnified FESEM images (Fig. 6b), Au NPs and Pt NPs exhibited a round (Fig. 6b-i) and sharp nanoflake (Fig. 6b-ii) shape, respectively. Upon the introduction of Au NPs into Pt NPs with a ratio of 1:4, the sharp nanoflake shape transformed to a nearly round shape (Fig. 6b-iii), presumably due to the lower surface energy of Au than for Pt^32^. To enhance the stimulation performance of the neural electrode, iridium oxide (IrOx) NPs were electro-deposited onto the already NP-modified electrodes. The elemental mapping for the IrOx/Au-Pt NP-modified electrode (Fig. 6c) revealed that both Au and Pt atoms are uniformly distributed in bimetallic Au-Pt NPs and the IrOx NPs are uniformly electro-deposited onto the bimetallic Au-Pt NP layer with an approximate thickness of 20 nm. As shown in Fig. 6d, the IrOx/Au-Pt NP-modified electrode exhibited the lowest impedance of 4.2 ± 0.15 kΩ at 1 kHz. As shown in Fig. 6e, the IrOx/Au-Pt NP-modified electrode exhibited the highest cathodic current density, yielding a cathodic charge storage capacity of 3.44 mC/cm^2^. Figure 6f. shows the voltage transient characteristics of the IrOx/Au-Pt NP-modified electrode as measured by applying cathode-first biphasic symmetric current pulses with a 100 µs pulse duration and 30 µs inter-delay between pulses. The charge injection limit of the IrOx/Au-Pt NP-modified electrode – derived from the voltage transient characteristics at different current amplitudes – was 0.55 mC/cm^2^, comparable to that of titanium nitride^33^.

## Discussion

The formation of SU-8 test patterns via conventional photolithography and the subsequent adhesion of SU-8 test patterns to fluoropolymers demonstrate simultaneous photoadhesion and photopatterning dual-function of photocrosslinkable polymers and the plasma-treated fluoropolymer system. The general applicability of the simultaneous P&P technique was confirmed by measuring the AS of some commercially available negative photoresists (Fig. 2g). The electrode passivation capability of P&P technique was confirmed by observed photopatterning fidelities for the SU-8 and FEP composite system, down to 20 μm-diameter patterns being photopatterned on both the FEP and the sputter-deposited Au layer (Fig. 3a,b). The advantages of our P&P technique include its simplicity in achieving robust adhesion between the passivation layer and the fluoropolymer substrate and the wide range of possible photocrosslinkable polymers and fluoropolymers. The AS data exhibiting an insensitivity to UV exposure time (Fig. 3c) proves that adhesion of SU-8 to a fluoropolymer is not governed by the number of photo-generated radicals within SU-8 bulk, yet by the radical density within the fluoropolymer. The photoadhesion of a photocrosslinkable polymer to a fluoropolymer can be classified as an extension of graft polymerisation which is a member of radical reactions typically utilised for surface modifications^34,35^.

The resemblance between the AS of SU-8 with fluoropolymers and the radical density of plasma-treated fluoropolymers with respect to the treatment time (Fig. 2a and b) indicates that the AS is closely related to the radical density. However, some discrepancies were observed. For example, the radical density of PFA is between that of FEP and PTFE, while the SU-8–PFA AS is close to that of FEP. According to Momose et al.^20^, the plasma treatment of PTFE followed by air-exposure generates both cross-linked peroxy radicals and chain scission peroxy radicals, verified by X-ray photoelectron spectroscopy and ESR spectroscopies. The cross-linked peroxy radical is the most abundant radical and is localised near the surface, yet the chain scission peroxy radical populates the bulk region. It has also been shown that the cross-linked peroxy radical exhibits a slower decay than the chain scission peroxy radical. In the cases of PFA and FEP, various radical groups can be generated due to side-chain moieties, and contribute to the observed radical density.

Considering the symmetric nature of the chain scission peroxy radical, the observed slightly asymmetric ESR spectrum shown in Supplementary Fig. 3 may reflect a mixed contribution of the various radical groups. The increased portion of chain scission radical would lead to formation of a weak boundary layer^36^, which in turn reduces AS. Therefore, to accurately derive the relationship between the AS and the radical density, it is reasonable to resolve the radical density to only those radicals which contribute to adhesion. The appearance of lamella structures after shorter plasma treatment times (Supplementary Fig. 4) indicates a preferential etching at the amorphous-to-crystalline region, resulting in an abrupt increase in both radical density and AS between SU-8 and fluoropolymer; presumably due to the increase in plasma-treated surface area. The subsequent appearance of nanoprotrusion structures and subsequent saturation in the radical density are attributed to the reduction in amorphous phase content, indicating an insignificant contribution to the SU-8–fluoropolymer AS from the roughened protrusion structure. Low AS values obtained at very low RF power (2–3 W) and very short treatment times (1–2 s), as shown in Fig. 3e, may imply that they are accomplished by partial surface coverage of plasma-generated radicals and not by the plasma etching-induced anchoring. This is further supported by the fact that the FESEM image of FEP plasma-treated at 2 W for 2 s did not exhibit the typical lamella structure. Assuming that the AS is proportional to the surface coverage of radicals, the measurement of the AS could be a useful tool in determining the surface coverage of radicals for studying the initial stage of plasma treatment of fluoropolymers.

The increased AS and radical density observed for FEP and PFA (Fig. 2a,b) implies that the AS is closely related to the radical density. Prevention of chain packing via the introduction of –CH_3_ and –OCF_3_ pendant groups on PTFE may facilitate the formation of additional radicals. The higher radical yield of γ–irradiated FEP^37^ and PFA^38^ (than that of PTFE) was previously reported; in those studies, this observation was attributed to both the greater amount of amorphous content and the scission at hexafluoropropylene and perfluoropropyl vinyl ether units. The limitation of cage recombination, due to higher chain mobility in the amorphous phase, is the suggested mechanism for the observed high radical yields for both FEP^37^ and PFA^38^. These results indicate that the crystallinity of the fluoropolymer is a crucial material factor for determining the AS between SU-8 and fluoropolymer. The as-polymerised FEP and PFA are known to possess a rough crystallinity of 70%, compared to 95–98% for the as-polymerised PTFE^39,40^.

Mechanical interlocking has been proposed as an underlying adhesion mechanism between noble metals and polymers with low surface energy. As mechanical interlocking can result from a roughened interface due to plasma etching and the formation of metal clusters^41^, the effect of plasma-induced roughness and metal clusters on the adhesion between Au and fluoropolymers was investigated. Contrary to the SU-8-fluoropolymer AS, the Au–fluoropolymer AS exhibited an insensitivity to the plasma treatment time, indicating an insignificant contribution of the conical nanoprotrusion structure to the adhesion between Au and fluoropolymer. It has been theoretically and experimentally verified that the energy influx from an impinging beam onto the substrate is maximised when the impinging angle is zero and decreases with respect to the cosine of this angle^42^. The steep slopes observed for the nanoprotrusions (Fig. 5a) would lower the energy influx, thereby mediating any increase in surface temperature and limiting both the penetration and diffusion of Au atoms into the peaks. Therefore, most impinging Au atoms migrate and aggregate to the valleys; this results in the enlarged clusters (Fig. 5a). A preferred etching of the amorphous-to-crystalline phase would contribute to the accumulation of Au clusters within the valleys. Although the enlarged clusters appear to help maintain interfacial adhesion, they are oriented toward the valleys in a manner preventing their contribution to adhesion for microscale patterned Au layer. Notably, the formation of deep conical protrusions is accompanied by an observable increase in the sheet resistance of Au. These results indicate that longer plasma treatments are not advantageous for the fabrication of neural electrode based on fluoropolymers.

The non-zero ASs between Au and non-treated fluoropolymers (Fig. 4a) are also quite different from that adhering SU-8 and fluoropolymers, which exhibited non-adhesion to non-treated fluoropolymers. The aforementioned non-zero ASs between Au and non-treated fluoropolymers coupled with the presence of Au clusters along the flat interface (Fig. 5a) indicate that the primary contribution of Au clusters to adhesion is through the mechanical interlocking mechanism. In previous studies examining the metallisation of polymers, the amount of metal atoms penetrating into the polymer bulk largely depends on both the surface temperature and the mobility of the polymer chain^43–45^. Therefore, considering the glass transition temperature (*T*_g_) of FEP (80 °C)^46^, PFA (90 °C)^47^, and PTFE (126 °C)^48^, that the ASs of Au to both FEP and PFA are nearly identical and that both are higher than that between Au and PTFE roughly correlated to the material *T*_g_ values. Under the assumption that the AS is closely related to the number of clusters, the substantially larger amount and deeper diffusion of Au clusters observed in both FEP and PFA compared to those of PTFE (Fig. 5b) support this interpretation. Additionally, these results and observations indicate that the crystallinity of a fluoropolymer significantly affects the adhesion of Au to a fluoropolymer, as a polymer’s *T*_g_ is highly influenced by the degree of crystallinity^49^. The higher AS of Au with thick fluoropolymer films (Fig. 4a) and its relationship to RF sputtering power (Fig. 4b) indicate that the factors affecting the surface temperatures – such as substrate thickness^50^ and RF sputtering power^51^ – are important for enhancing the adhesion between Au and fluoropolymers. For example, thicker substrates offer an increased thermal resistance for heat transfer from the substrate surface to metallic substrate holder, leading to an increased substrate surface temperature, as well as an increased penetration and diffusion of impinging metal atoms into the substrate. For thinner films, the additional substrate heating would induce deeper penetration of Au atoms, leading to an enhanced adhesion via an increased cluster formation probability.

The successful fabrication of a 16-CH ECoG Au electrode array based on SU-8 and a 127 m-thick FEP substrate demonstrates the passivation ability of the proposed simultaneous P&P technique and the adhesion enhancement ability of an RF magnetron sputter deposition of Au onto fluoropolymer. Additionally, successful surface modifications on the Au electrode with Au, Pt, and Au-Pt metallic NPs by electrodeposition demonstrates the passivation feasibilty of the photoadhered SU-8 layer to the FEP substrate. A CSC of 2.63 mC/cm^2^ and a charge injection limit of 0.55 mC/cm^2^ were obtained from the IrO_x_/Au-Pt NP-modified electrode, indicating the synergetic role of IrO_x_ with Au-Pt NPs. Although we have fabricated an ECoG electrode array through a SU-8 and FEP composite for demonstration purposes, the proposed simultaneous P&P technique can be applied to a wide range of flexible devices that require chemically robust passivation, such as implantable electrochemical sensors, biosensors, wearable sensing devices, and electronic skin devices. Recently, long-lived organic radical species have attracted much attention owing to their unique electronic and magnetic properties^52^; therefore, our P&P technique may pave the way for new functional device fabrication by offering a robust adhesive interface with fluoropolymers.

## Methods

### Pull-off test

FEP, PFA, and PTFE films from CS Hyde were used as a substrate for our photoadhesion studies. Prior to any plasma treatment, the fluoropolymer films were cleaned with ethanol under sonication for 1 min and were then rinsed with deionised water followed by drying with nitrogen gas. The cleaned fluoropolymer film surface was covered with a metal shadow mask possessing an 8 mm-diameter opening; the opened surface was then treated with RF magnetron Ar plasma to generate radicals. An in-house fabricated plasma system equipped with an 8″ RF magnetron gun from Angstrom was used for the plasma treatment. Typically, Ar gas was supplied at a maintained pressure of 15 mTorr during plasma treatment. SU-8 3010 (MicroChem) was spin-coated onto the plasma-treated fluoropolymers and subsequently prebaked at 95 °C for 3 min. The sample was then exposed to a ultraviolet light-emitting diode array (22.7 mW/cm^2^ @ 365 nm, MIDAS, Korea) through an 8 mm-diameter blank patterned on a chromium photomask typically for 8 s, followed by a 3 min post-exposure bake at 95 °C. Samples were then immersed into the SU-8 developer (MicroChem) for 8 min followed by a rinse with isopropyl alcohol. After drying under nitrogen gas, an 8 mm-diameter metal stud was adhered to the SU-8 test pattern with two-component epoxy. The epoxy was hardened on a hot plate maintained at 60 °C for 90 min. The adhesive strength measurement was performed by a z-axis pull-off test^53^ using a commercially available tensile tester (BMS Tech, Korea). The pulling speed of the stud was fixed at 2 mm/min. Commercially available negative-tone photoresists such as LTC 9520 (FujiFilm), AZ nLOF 2070 (AZ), and NR9-3000PY (Futurrex) were applied as the photocrosslinkable polymer and photopatterned according to technical guides supplied by manufacturers.

To measure the AS between Au and a fluoropolymer, fluoropolymer film was screened with a thin metal mask possessing an 8 mm-diameter opening; the opened surface was plasma treated and sputter-deposited with a 200 nm-thick Au and a 2 μm-thick Al. Because of the inherently weak adhesion between epoxy and Au, the thick Al layer was introduced as an adhesion promotor. An in-house fabricated RF (13.56 MHz) magnetron sputter system was used for Au deposition. Typical sputtering conditions are: Ar at a working pressure of 5 mTorr and RF power of 50 W. All AS data were averaged from 8 to 16 trials after the removal of the highest and lowest values.

### Surface and interface characterisations

The plasma-generated radical density of the plasma-treated 254 μm–thick fluoropolymer was estimated from the number of radicals calculated by double integration of the ESR signal intensity after indexing for peroxy radicals. An ESR spectrometer JES-FA100 (JEOL) equipped with an X-band source was used. ESR spectra were obtained within 20 min of plasma treatment; these measurements were made at room temperature, with a microwave power of 1 mW, and at an applied frequency of 9.1 GHz. The sample was 3 mm × 30 mm in size, cut out from the 254 μm–thick film after plasma treatment. The radical density was calculated from the signal intensity after having been calibrated in a solution of 1 × 10^–5^ mol 2,2,6,6-tetramethyl-1-piperidinyloxyl. The surface morphology of the plasma-treated fluoropolymers, the micro-photopatterns, and the surface morphology of the metallic NP-modified Au electrode were imaged using a FESEM (SU5000, Hitachi). The cross-section of the Au-FEP interface and that of the metallic NP-modified electrode were analysed using two field emission transmission electron microscopes (JEMARM200 F; JEOL, TENNAI F30; FEI). The detailed compositional distribution of both Au and Pt in the Au-Pt bimetallic NPs and the presence of the electro-deposited ultrathin thin IrOx layer were confirmed by analysing results from scanning transmission electron microscopy – energy-dispersive X-ray spectroscopy (STEM-EDS) elemental mapping images.

### ECoG MEA fabrication

For this fabrication the 127 μm–thick FEP film was cleaned via plasma treatment at 20 W for 1 min. After cleaning, a 1 μm–thick Au layer was deposited by two-step RF magnetron sputtering (100 W for 40 s and 50 W for 1 h). The samples then underwent patterning to have a 4 × 4 electrode array, interconnecting lines, and connecting pads through both conventional photolithography and wet Au etching using Au etchant (GOLD ETCH – type TFA, Transene); samples were then plasma-treated at 20 W for 1 min. Then, SU-8 3010 was spin-coated and photopatterned as described within the tensile strength measurement section. The diameters of the opened-electrodes were 25, 50, and 100 μm.

### Electrochemical surface modification

Gold (III) chloride trihydrate (HAuCl_4_), chloroplatinic acid hexahydrate (H_2_PtCl_6_), potassium ferricyanide (III) (K_3_Fe(CN)_6_), and potassium hexacyanoferrate (II) trihydrate (K_4_Fe(CN)_6_) were purchased from Aldrich. Iridium chloride (IrCl_4_), potassium carbonate, and oxalic acid were purchased from Alfa Aesar and hydrogen peroxide (H_2_O_2_) was purchased from Junsei. All chemicals were used as received without additional purification. The iridium oxide (IrO_x_) solution was prepared according to the report by Hu et al.^54^. The conventional three-electrode configuration was used in both electrodeposition and electrochemical measurements. The electrode in the flexible ECoG electrode array, a Pt plate, and Ag/AgCl was used as a working, counter, and reference electrode, respectively. The working electrode was connected to a ModuLab Femtostat system (Solartron Analytical) via a 16 CH Omnetics connector. All potentials reported are against the Ag/AgCl electrode submerged in a saturated potassium chloride solution.

The electrodeposition of metallic NPs was carried out by the potentiostatic method at -0.2 V. Au and Pt NPs were electrodeposited from solutions of 1 mM HAuCl_4_ and 4 mM H_2_PtCl_6_, respectively; they were then electro-co-deposited in a mixture of the two solutions. IrOx NPs were electrodeposited on metallic NP-modified electrodes by the CV method with a potential range of -0.5 V ~ 0.7 V and at a scan rate of 100 mV/s. Electrochemical impedance spectroscopy, CV, and voltage transient measurements were performed in a 0.1 M KCl solution containing 2.5 mM 1:1 K_4_Fe(CN)_6_/K_3_Fe(CN)_6_.

## Supplementary information

Supplementary Information.

## Data Availability

All data generated and/or analysed during this study are included in this article and Supplementary Information file and available from the corresponding author on request.
