# Supplementary material for: Simultaneous photoadhesion and photopatterning technique for passivation of flexible neural electrodes based on fluoropolymers
Source: Sci Rep. 2020 Dec 7;10:21386. doi: 10.1038/s41598-020-78494-w (PMC7721713; doi:10.1038/s41598-020-78494-w)
Supplement: Supplementary file 1 — Supplementary Information. [file 41598_2020_78494_MOESM1_ESM.docx]

**Supplementary Materials for**

**Simultaneous Photoadhesion and Photopatterning Technique for Passivation of Flexible Neural Electrodes Based on Fluoropolymers**

Yong Hee Kim^1^ & Sang-Don Jung^1^*

^1^ ICT Creative Research Division, Electronics & Telecommunications Research Institute, 218 Gajeong-ro, Yuseong-gu, Daejeon 34129, Republic of Korea

*Corresponding author: jungpol@etri.re.kr

**
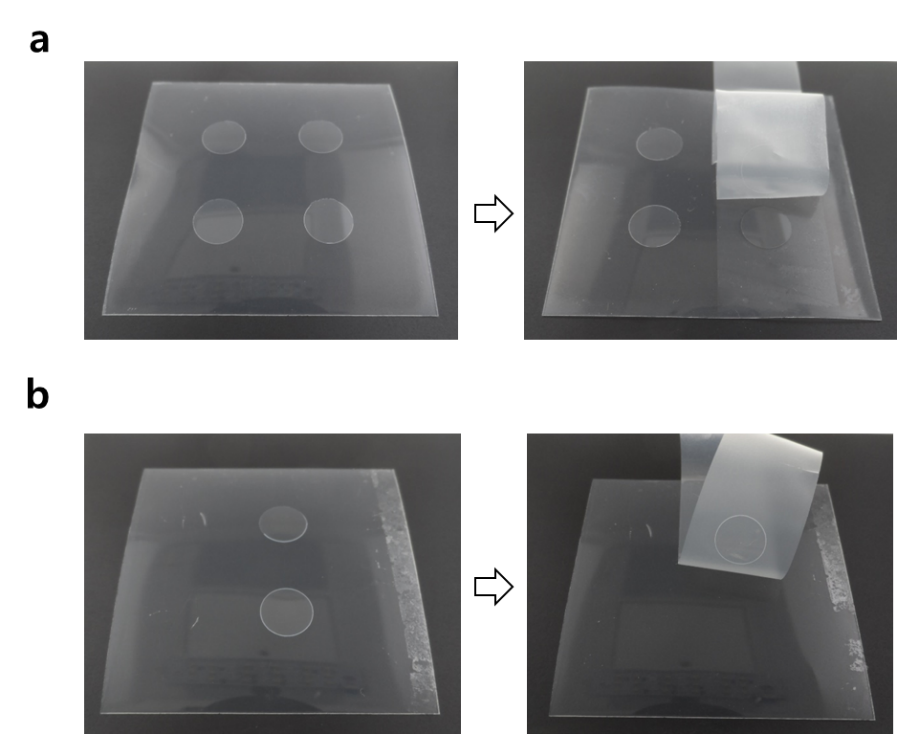
**

**Supplementary Figure S1. .** Photographs of (**a**) SU-8 test patterns photopatterned on plasma-treated FEP (RF power: 40 W, treatment time: 4 min) before (left) and after (right) tape peel test and (**b**) SU-8 test patterns photopatterned on non-treated FEP before (left) and after (right) tape peel test. It should be noted that SU-8 is uniformly spin-coated on the plasma-treated FEP but not on the non-treated FEP film. The scotch^®^ tape (810D) was rubbed with 2-Kg standard roller.

**
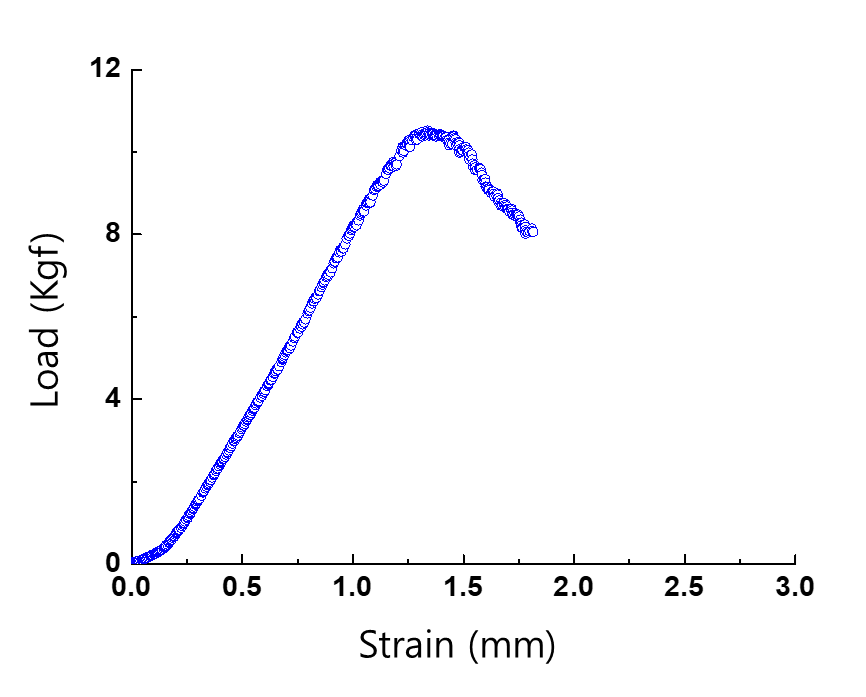
**

**Supplementary Figure S2.** Typical load-strain curve for a SU-8 test pattern photopatterned on plasma-treated FEP (RF power; 10 W, treatment time; 1 m). The pull speed was 2 mm/min.


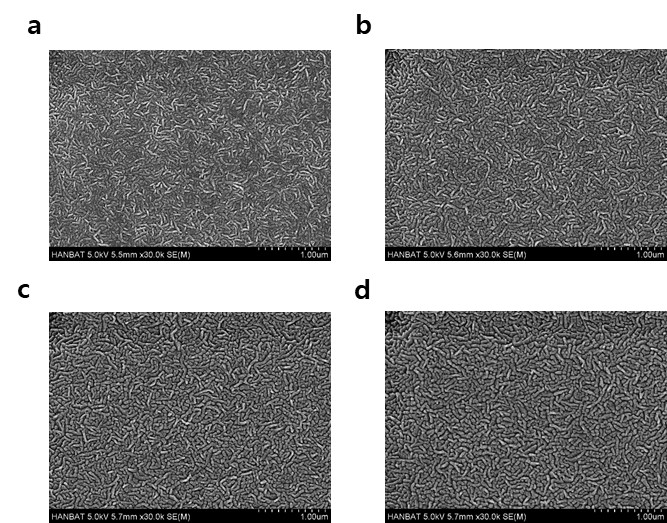


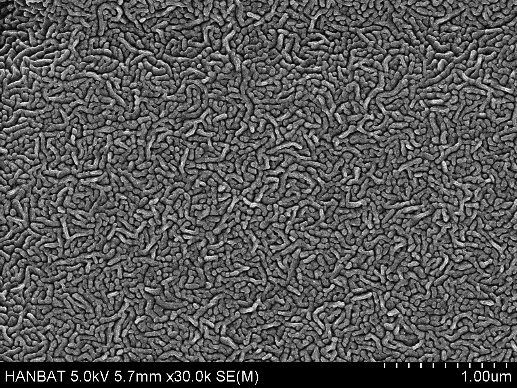


**Supplementary Figure S3.** FESEM image of FEP Ar RF plasma-treated at 40 W for (a) 15, (b) 30 s, (c) 45 s, and (d) 1 min.

**
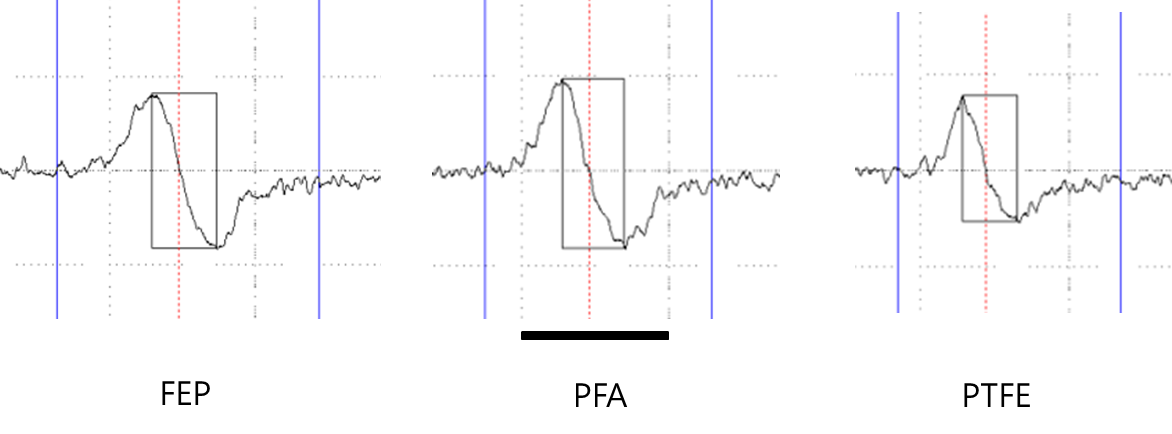
**

**Supplementary Figure S4.** Typical ESR spectra for FEP (left), PFA (middle), and PTFE (right). X-axis and Y-axis denotes magnetic field (mT) and intensity (a.u.), respectively. The scale bar denotes 5 mT.


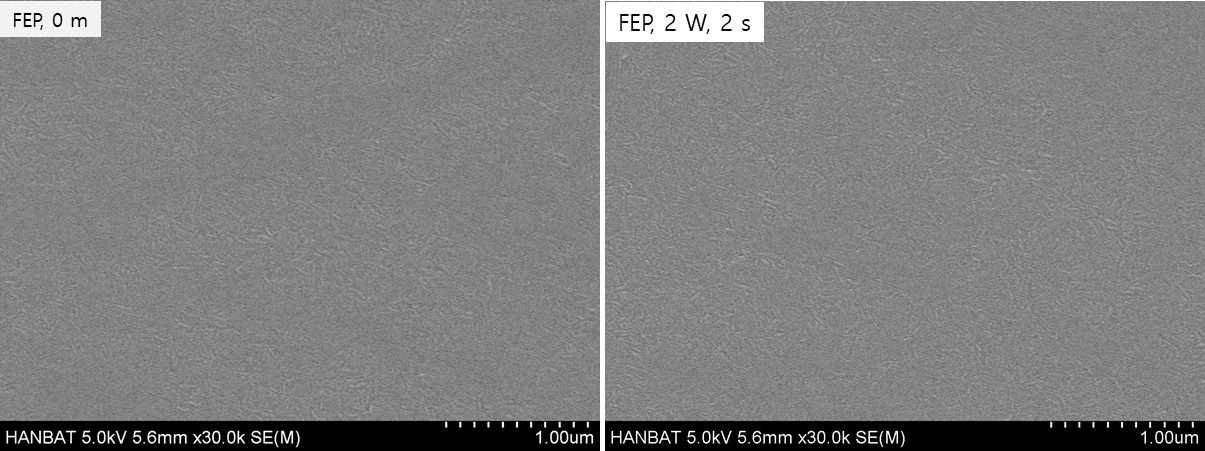


**Supplementary Figure S5.** FESEM image of intact FEP surface (left) and plasma-treated FEP surface (right); RF power: 2 W and treatment time: 2 s.


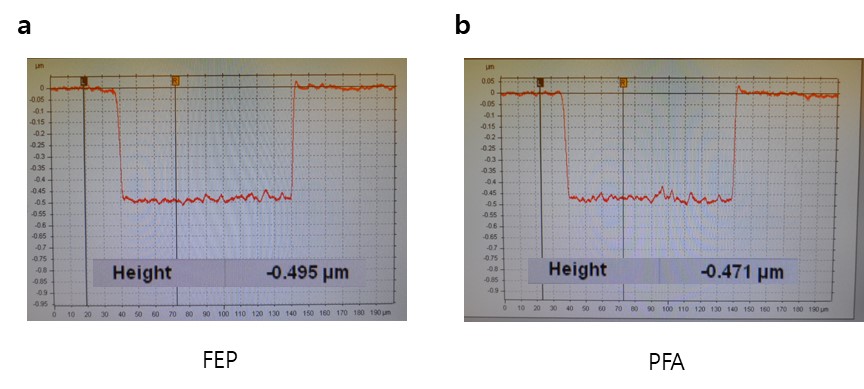


**Supplementary Figure S6.** Etched depth profile of Ar RF plasma-treated (a) FEP and (b) PFA (RF power: 40 W, working pressure: 15 mTorr, treatment time 8 min). In case of PTFE the clear etched depth profile could not be obtained due to rough surface profile of intact PTFE.

**Fabrication.** We thermally deposited aluminium (Al) on the 127-μm thick FEP and PFA films and patterned both line and spacing patterns via both conventional photolithography and wet etching. The etch depth was also examined for Ar plasma-treated (RF power: 40 W, working pressure: 15 mTorr, treatment time: 8 min) FEP and PFA films using a profilometer after removing the aluminium patterns using Al etchant.


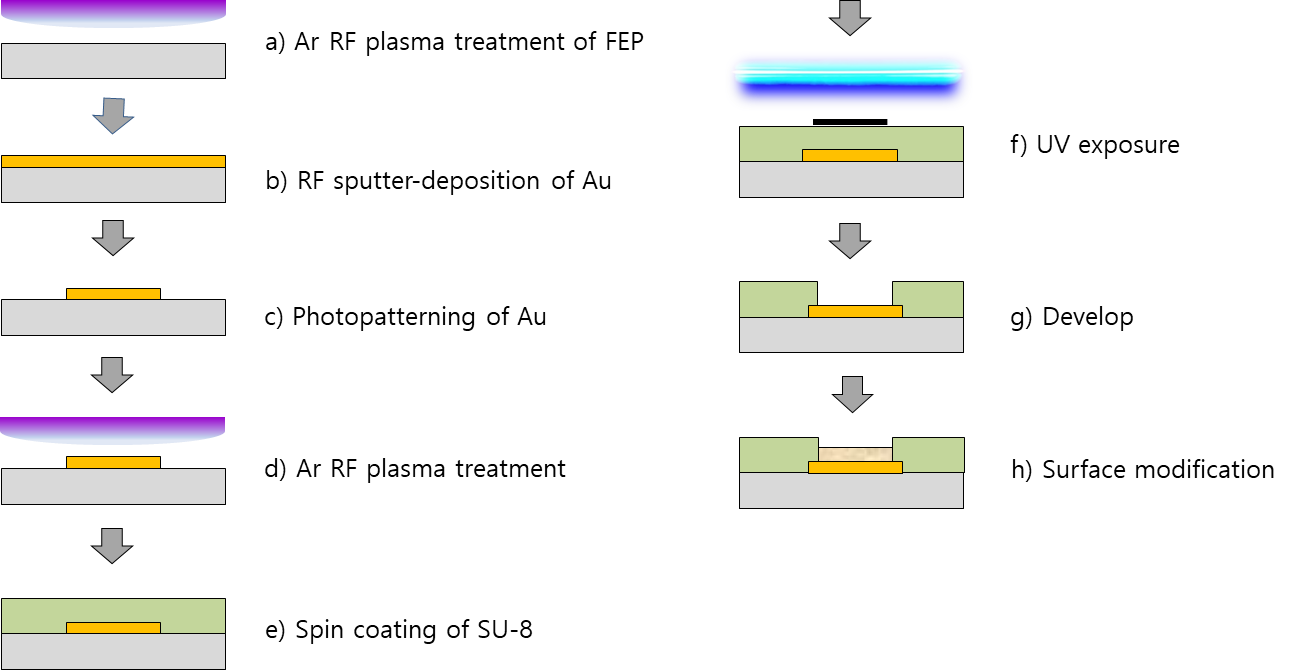


**Supplementary Figure S7.** Schematic representation of an ECoG electrode array fabrication process based on fluoropolymer and the proposed simultaneous P&P technique using FEP film and SU-8 as a substrate and passivation layer, respectively.

**Supplementary Table T1.** Tensile modulus of FEP, PFA, and PTFE^§^.

|  | Tensile modulus (MPa) |
| --- | --- |
| FEP | 520-600 |
| PFA | 435-530 |
| PTFE | 480-630 |

§: Data from Polymer Properties Database, https://polymerdatabase.com/
